# Role of Pannexin-1 hemichannels and purinergic receptors in the pathogenesis of human diseases

**DOI:** 10.3389/fphys.2014.00096

**Published:** 2014-03-14

**Authors:** Stephani Velasquez, Eliseo A. Eugenin

**Affiliations:** ^1^Public Health Research Institute, Rutgers the State University of New JerseyNewark, NJ, USA; ^2^Department of Microbiology and Molecular Genetics, Rutgers New Jersey Medical School, Rutgers the State University of New JerseyNewark, NJ, USA

**Keywords:** HIV, inflammation, connexins, atherosclerosis, ATP

## Abstract

In the last decade several groups have determined the key role of hemichannels formed by pannexins or connexins, extracellular ATP and purinergic receptors in physiological and pathological conditions. Our work and the work of others, indicate that the opening of Pannexin-1 hemichannels and activation of purinergic receptors by extracellular ATP is essential for HIV infection, cellular migration, inflammation, atherosclerosis, stroke, and apoptosis. Thus, this review discusses the importance of purinergic receptors, Panx-1 hemichannels and extracellular ATP in the pathogenesis of several human diseases and their potential use to design novel therapeutic approaches.

## Introduction

In recent years it has become evident that pannexin (Panx)-1 hemichannels in concert with extracellular adenosine triphosphate (ATP) and purinergic receptors are involved in several physiological and pathological conditions. Recent evidence suggests the participation of Panx-1 hemichannels, extracellular ATP and purinergic receptors in the coordination of events such as cellular activation, apoptosis, stress signals, secretion of inflammatory cytokines, and HIV replication have been explored (Pelegrin, 2008; Schenk et al., 2008; Chekeni et al., 2010; Woehrle et al., 2010; Qu et al., 2011; Seror et al., 2011; Orellana et al., 2013). This review will describe these interactions in the context of several human diseases.

The Panx family consists of three members, Panx-1, -2, and -3 (Baranova et al., 2004). Panx-1 is ubiquitously expressed (Scemes et al., 2009). Panx-2 is mainly expressed in the central nervous system (CNS), while Panx-3 is localized in osteoblasts, synovial fibroblasts and chondrocytes (Barbe et al., 2006; Ray et al., 2006). Originally, it was speculated that Panx hemichannels could form gap junction channels between adjacent cells (Bruzzone et al., 2003, 2005; Bruzzone and Dermietzel, 2006). However, current evidence suggests that Panxs cannot form intercellular channels (Boassa et al., 2007). Asparagine residues found on the extracellular domains of Panx are glycosylated and therefore make docking between two Panxs highly unlikely (Boassa et al., 2007; Penuela et al., 2007).

Panxs are structurally similar to connexins (Cxs), although they share no sequence homology. Panx consist of a cytosolic N-terminal domain, four transmembrane domains with two extracellular loops and a cytosolic C-terminal domain (Boassa et al., 2007; Penuela et al., 2007). Panxs form large pore channels located on the plasma membrane, which are known to open during membrane depolarization, by changes in intracellular Ca^2+^ signaling, vasodilation, vasoconstriction, taste sensation, airway defense, learning/memory, cellular differentiation, cell death and during innate, and adaptive immune responses (Chekeni et al., 2010; MacVicar and Thompson, 2010; Prochnow et al., 2012). Upon opening of these hemichannels small signaling molecules such as ATP are released to the extracellular space, which then signal through surface receptors including purinergic receptors.

Purinergic receptors are divided into two groups, Adenosine receptors (ARs) for adenosine and P2 receptors for ATP/ADP receptors (Fredholm et al., 1994; Ralevic and Burnstock, 1998). P2 receptors are subdivided into ionotropic P2X and metabotropic P2Y receptors. P2X receptors are ligand gated ion channels that form trimeric structures using individual subunits (P2X1, P2X2, P2X3, P2X4, P2X5, P2X6, and P2X7) (Fredholm et al., 1994; Ralevic and Burnstock, 1998). P2Y receptors are G-protein coupled receptors with eight subtypes (P2Y1, P2Y2, P2Y4, P2Y6, P2Y11, P2Y12, P2Y13, P2Y14). Purinergic receptor signaling is fundamental in many cellular processes such as platelet aggregation, exocrine and endocrine secretion, endothelial-mediated vasodilation, nociceptive mechanosensory transduction, neuromodulation, neuroprotection, cell proliferation, differentiation, migration, embryological development, wound healing, inflammation, and cytokine secretion (Abbracchio and Burnstock, 1998; Burnstock and Knight, 2004; Burnstock and Verkhratsky, 2010; Burnstock, 2012). Upon release of ATP into the extracellular space, several enzymes degrade ATP into ADP, AMP and adenosine, which also signals through purinergic receptors and adenosine receptors.

**Figure 1:**
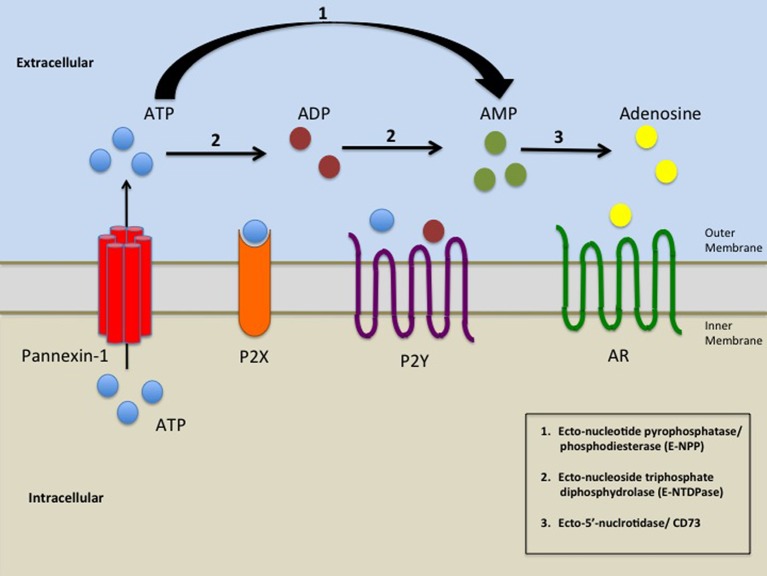
**A schematic representation of the elements involved in the release of ATP by opening of Panx-1 hemichannels and subsequent activation of purinergic signaling**. Pathological or physiological stimuli result in the opening of Panx-1 hemichannels promoting the release of ATP from the cell. ATP/ADP/AMP could then bind to P2X and P2Y receptors. Ecto-nucleoside triphosphate diphosphydrolase (E-NTDPase) including ecto-ATPase and ATP-diphospho-hydrolase promotes the hydrolysis of ATP to ADP or from ADP to AMP (2). Ecto-nucleotide pyrophosphatase/phosphodiesterase (E-NPP) hydrolysis ATP to AMP (1). AMP is further hydrolyzed by Ecto-5'-nucleotidase/ CD73 (3) which promotes the formation of adenosine. Adenosine then activates adenosine receptors (AR).

ARs (A1, A2A, A2B, and A3) were initially classified as P1 receptors until it was discovered that their agonist was adenosine (Fredholm et al., 1994; Junger, 2011). As indicated above, the production of extracellular adenosine is achieved by hydrolyzing ATP in a stepwise manner by ectonucleotidases (Yegutkin, 2008). These enzymes include the Ecto-nucleotide pyrophosphatase/phosphodiesterase (E-NPP) family comprised of three enzyme subtypes, which hydrolyses ATP to AMP, and Ecto-nucleoside triphosphate diphosphydrolase (E-NTDPase) family comprised of eight enzyme subtypes, which can hydrolyze ATP to ADP or AMP. Finally, Ecto-5'-nucleotidase/ CD73 in tandem with CD38 can hydrolyze AMP to adenosine, which then activates the ARs (Yegutkin, 2008; Junger, 2011). In Figure 1 we summarized the interaction between Panx-1 hemichannels, purinergic receptors, adenosine receptor, as well as the extracellular metabolism of ATP (Figure 1). In the next sections we will discuss the involvement of this complex in several human diseases.

## Purinergic receptors, Panx-1 hemichannels and their involvement in ischemic stroke

According to the World Health Organization (WHO), 15 million people suffer stroke worldwide each year, resulting in 5 million deaths and another 5 million survivors that are permanently disabled (www.WHO.int). Ischemic stroke results from a permanent or transient decrease in cerebral blood flow. This decrease in blood flow is usually caused by the obstruction of a cerebral artery by an embolus or local thrombosis (Katsura et al., 1994; Martin et al., 1994; Dirnagl et al., 1999). Brain tissue requires a high intake of glucose and oxygenation for proper cerebral function. The restriction of cerebral blood flow impairs the delivery of glucose and oxygen and consequently leads to tissue damage by mechanisms dependent on excitotoxicity, peri-infarct depolarizations, inflammation and programmed cell death (Katsura et al., 1994; Martin et al., 1994; Dirnagl et al., 1999).

Thompson et al. demonstrated a connection between Panx-1 hemichannels and ischemia using acutely isolated hippocampal neurons in which oxygen and glucose deprivation (OGD) resulted in opening of Panx-1 hemichannels (Thompson et al., 2006). Blocking NMDA (N-methyl-D-aspartate), AMPA (2-amino-3-[5-methyl-3-oxo-1,2-oxazol-4-yl] propanoic acid) and P2X7 receptors failed to modify the large anoxic depolarization activated by OGD, which corresponded to opening of Panx-1 hemichannels. Therefore, the mechanism by which Panx-1 hemichannels are opened during OGD was thought to be independent from ligand-gated receptors (Thompson et al., 2006). Recent evidence suggests that anoxia induces NMDA receptor activation, which activates Src kinases, that participates in the opening of Panx-1 hemichannels (Weilinger et al., 2012). This suggests a signaling pathway involving the coupling of NMDA receptors using Src Kinases to Panx-1 hemichannels. Furthermore overstimulation of NMDA receptors activates the opening of Panx-1 hemichannels in neurons (Thompson et al., 2008). However, another study demonstrated that inhibiting glutamate receptors of hippocampal pyramidal slices prevented anoxic depolarization and that Panx1 hemichannels did not generate a large inward current associated with anoxic depolarization (Madry et al., 2010). Therefore, future studies are required to clarify the participation of Panx-1 hemichannels in response to anoxia/ischemia.

ATP is a neurotransmitter that mediates communication between CNS cells, including astrocytes and neurons. Initially, it was believed that the mechanism by which ATP was released from astrocytes involved Connexin43 (Cx43) hemichannels. However, experiments conducted using wild type, Cx43 null, and Panx-1 knocked down astrocytes provided evidence indicating that downregulation of Panx-1 prevented the release of ATP from astrocytes (Iglesias et al., 2009). Downregulation of Cx43 has no affect on the release of ATP from astrocytes. In contrast, experiments using conditional Cx43 knockout demonstrated that ATP release and recruitment of microglia/macrophages following injury was reduced and the recovery of the animals was improved, suggesting a role for Cx43 hemichannels in inflammation but also in recovery (Huang et al., 2012). Orellana et al. demonstrated that under hypoxic conditions astrocytes release ATP and glutamate activating neuronal Panx-1 hemichannels via P2X and NMDA receptors resulting in neuronal death (Orellana et al., 2011). This study demonstrates that neurons could be protected from ischemia-associated damage by blocking NMDA/P2X receptors as well as Panx-1 hemichannels (Orellana et al., 2011).

Furthermore experiments conducted in the double Panx-1 and Panx-2 knockout mice subjected to permanent right middle cerebral artery occlusion (MCAO) demonstrated that Panx channels contributed to ischemic brain injury *in vivo* (Bargiotas et al., 2011). The double knockout mice subjected to MCAO demonstrated improved neurological deficit, reduced movement latency and infarct size as compared to the wild type (Bargiotas et al., 2011). Single knockouts of either Panx-1 or Panx-2 did not differ in ischemic brain injury from wild type; however, Panx-2 knockout mice were partially protected from ischemic injury. These data suggests that Panx-1 and Panx-2 work together to regulate response to injury.

Ischemia induces astrocytes to release ATP, which rapidly activates microglia resulting in the formation of a barrier between the healthy and injured tissue in order to promote repair (Davalos et al., 2005; Nimmerjahn et al., 2005). Moreover, an excess release of nucleotides can result in accelerated neurodegeneration (Di Virgilio et al., 2009). An excessive level of extracellular ATP in oligodendrocytes induces a rise in cytosolic Ca^2+^ by activating P2 receptors and P2Y7 receptors (Kirischuk et al., 1995; James and Butt, 2001). Using primary cultures of oligodendrocytes it was demonstrated that OGD induced the release of ATP and blocking of P2X7 receptors using periodate oxidized ATP (oATP) or Brilliant Blue G (BBG) reduces the ischemic-induced ionic imbalance. In addition, reduction in opening of Panx hemichannels using blockers such as mefloquine and flufenamic acid reduced extracellular ATP levels after OGD attenuating ischemic damage. These data indicates that OGD opens Panx hemichannels inducing the release of ATP which then activates P2X7 receptors causing oligodendrocytes failure, myelin damage and axon dysfunction (Domercq et al., 2010).

Furthermore, elevations in the expression of several P2 receptors (P2X1, P2X2, P2X4, P2X7, and P2Y4) during ischemia have been demonstrated suggesting increased sensitivity of neurons to extracellular concentration of ATP (Cavaliere et al., 2002, 2003, 2007). Using spontaneously hypertensive rats (SHR) subjected to MCAO Lammer et al. demonstrated that inhibition of P2 receptors by pyridoxalphosphate-6-azophenyl-2', 4'-disulfonate (PPADS) improved the recovery of the cortical electrophysiological and motor functions (Lammer et al., 2006, 2011). PPADS does not pass through the blood brain barrier; therefore, the rats were infused by intracerebroventricular administration for 7 days after MCAO. Furthermore, analysis of motor coordination demonstrated that blockade of P2 receptors by PPADS resulted in improved motor recovery when compared to non-PPADS treated rats subjected to MCAO (Lammer et al., 2011). Thus, opening of Panx-1 hemichannels and activation of P2 receptors play a major role in the pathogenesis of ischemia and blocking or knocking down these hemichannels/receptors could provide additional therapeutic interventions to reduce damage and to improve recovery in response to ischemic events.

## Panx hemichannels, purinergic receptors and inflammation

Tissue damage causes the release of ATP from injured cells, resulting in P2 receptor mediated purinergic signaling and the initiation of inflammation (Bours et al., 2006; Kanneganti et al., 2006; Mariathasan et al., 2006). During this process in both immune and parenchymal cells, hemichannels are open in concert with activation of purinergic receptors to control cellular migration, inflammation, and damage.

As indicated above an essential aspect of inflammation is the migration of inflammatory cells into areas of injury. Cellular migration requires mechanisms to allow orientated movement including sensing changes in the chemoattractant gradient, activation of G-protein coupled receptors, and downstream signaling resulting in cytoskeletal rearrangement leading to movement toward the chemotactic signal. Recent evidence suggests that Panx-1 hemichannels and P2X7 could initiate an intracellular signaling cascade which, results in rearrangement of the F-actin microfilament network in C6 glioma cells causing the assembly of large tumor cell aggregates (Bao et al., 2012). A similar actin microfilament rearrangement as mentioned above is a critical step in cellular migration. Intracellular ATP is released through Panx-1 hemichannels and then binds to the P2X7 receptor (see Figure 1), which causes an increase in intracellular calcium resulting in actin microfilament organization (Cotrina et al., 1998; Suadicani et al., 2006). Current evidence suggests that the release of ATP by a Panx-1 hemichannel mediated mechanism from apoptotic cells function as “Find me signals” in order to recruit monocytes to areas of damage (Chekeni et al., 2010). Our laboratory demonstrated that chemokines that bind to CCR5 or CXCR4 transiently open Panx-1 hemichannels in T lymphocytes, suggesting that these channels also play a key role in surveillance and inflammation. Thus our work and the work of others propose that Panx-1 hemichannels, extracellular ATP and purinergic receptors are essential in immune surveillance and inflammation.

Migration in a chemotactic gradient requires excitatory signals at the front of the cell and inhibitory signals at the back of the cell (Berzat and Hall, 2010). In this context ATP released by Panx-1 hemichannels stimulates the P2Y2 receptors, which provides the excitatory signal at the front of the cell (Chen et al., 2006, 2010). Bao et al. demonstrated that Panx-1 hemichannels provides the ligand for the adenosine A2A receptors that plays a role in the inhibitory signal at the back of the cell (Bao et al., 2013). Resting neutrophils had a uniform distribution across the cell of A2A receptors and polarized cells had the A2A receptors redistributed to the back of the cell where they provided the inhibitory signal. Inhibition of Panx-1 hemichannels blocked A2A receptor stimulation preventing the accumulation of cAMP, impairing the polarization and migration of neutrophils in a chemotactic gradient (Bao et al., 2013). These results suggest that chemoattractant receptors require opening of Panx-1 hemichannels in order to provide excitatory and inhibitory signals for efficient chemotaxis of neutrophils.

Inflammasomes are large multiprotein complexes leading to caspases-1-activated maturation of IL-1β and IL-18. The NLRP3 inflammasome is the most studied inflammasome containing NLRP3 as a scaffold protein (Schroder and Tschopp, 2010; Davis et al., 2011). NLRP3 inflammasomes are activated via danger-associated molecular patterns (DAMPs) such as extracellular ATP, which act through P2X7 receptors (Lich et al., 2006; Meylan et al., 2006; Said-Sadier and Ojcius, 2012). There are several proposed mechanisms, which induce NLRP3 inflammasome activation such as, reactive oxygen species (ROS) production and apoptosis (Said-Sadier and Ojcius, 2012). Cell induced ROS production and immune activation, have been shown to induce caspase-1 activation (Cruz et al., 2007; Said-Sadier and Ojcius, 2012). Hung et al. demonstrated that activation of P2X4 and P2X7 in response to ATP released by Panx-1 hemichannels contributed to ATP induced ROS production and inflammasome activation in gingival epithelial cells (Hung et al., 2013). Inhibitors of P2X4, P2X7, and Panx-1 significantly reduced ATP dependent production of ROS. Reduced expression of P2X4, P2X7, and Panx-1 using siRNA demonstrated that both purinergic receptors and Panx-1 hemichannel were required for ATP-induced ROS production in primary and immortalized gingival epithelial cells (Hung et al., 2013). Furthermore, recent evidence identifies that the NLRP3 inflammasome is activated during the phagocytosis of dying autophagic cells. This mechanism involves the release of ATP through Panx-1 hemichannels of the dying autophagic cell, P2X7 receptor activation and potassium efflux (Ayna et al., 2012).

In agreement, patients who have chronic lung inflammation such as allergic asthma or chronic obstructive pulmonary disease have enhanced extracellular ATP levels in the bronchoalveolar space, as well as in the bronchoalveolar lavage fluids (BALF) suggesting that the enhanced lung inflammation observed in these individuals may be associated with ATP dysregulation and purinergic receptor activation (Idzko et al., 2007; Lommatzsch et al., 2010). Furthermore, P2X7 receptor deficient mice have been shown to have less neutrophil airway influx and Panx-1 hemichannel inhibitors partially prevent further neutrophil airway influx and cytokine production (Riteau et al., 2010). Extracellular ATP serves as a danger signal to the immune system by binding to P2X7 receptor and activating NALP3 and caspase-1 which then leads to the maturation and release of IL-1β, eventually forming the NALP3 inflammasome (Ferrari et al., 2006; Kanneganti et al., 2006; Mariathasan et al., 2006; Sutterwala et al., 2006; Di Virgilio, 2007). Extracellular ATP induced caspase-1 activation and IL-1β maturation requires P2X7 receptor and Panx-1 hemichannel (Pelegrin and Surprenant, 2006; Locovei et al., 2007). These data further suggests the involvement of Panx-1 hemichannels, purinergic receptors and extracellular ATP in inflammasome activation.

Another human disease involving Panx-1 hemichannels is inflammatory bowel diseases (IBD) including ulcerative colitis, and Crohn's disease. These diseases are chronic conditions associated with gut dysfunction resulting from alterations in the enteric nervous system leading to severe symptoms (Mawe et al., 2009). Currently not much is known about the expression of Panx-1 in the intestines. However, one study showed the expression of innexins in the gut of nematodes, which were needed for gut motility (Peters et al., 2007). Recently Gulbransen et al. showed using a mouse model of colitis that Panx-1 hemichannels are required for P2X7 receptor mediated enteric neuron cell death in intestinal inflammation (Gulbransen et al., 2012). They identified that Panx-1 hemichannels play a key role in enteric neuronal damage, leading to organ dysfunction. Inhibition of Panx-1 hemichannels protects neurons and maintains proper control of the colonic muscles preserving motility (Gulbransen et al., 2012). Diezmos et al. described the expression of Panx-1 in the human colon; they also described alterations in the expression of Panx-1 in IBD patients (Diezmos et al., 2013). Panx-1 mRNA and protein was present in all layers of the human colon. There was also dense expression of Panx-1 on the submucosal and myenteric ganglia further confirming the involvement of Panx-1 in neural control of colonic motility (Diezmos et al., 2013). These findings suggest a critical role of Panx-1 hemichannels in the pathophysiology of enteric plexus damage during IBD.

As described above ATP release by opening of Panx-1 hemichannels results not only in activation of ATP receptors but also in degradation of ATP to ADP, AMP, and adenosine. CD39 and CD73 are ectonucleotidases, which degrade ATP, ADP, and AMP to adenosine (see Figure 1). These ectonucleotidases play an essential role in maintaining immune homeostasis. Regulatory T cells (Tregs) are mediators of inflammatory response. High levels of CD39 and CD73 are expressed on the surface of Foxp3^+^ Treg cells (Mandapathil et al., 2009; Schuler et al., 2011). Murine Treg cells increase CD39 activity after activation of the T cell receptor and non-activated cells had inactive CD39 (Borsellino et al., 2007). Adenosine derived from the enzymatic breakdown of ATP by CD39 and CD73 mediates a considerable portion of the anti-inflammatory activities of Treg cells (Deaglio et al., 2007). Romio et al. showed that adenosine produced by Treg cells in concert with A2A receptors downregulated nuclear factor- κB (NF- κB) activation in T effector cells, which in turn reduced the release of proinflammatory cytokines and chemokines (Romio et al., 2011). Activation of the A2A receptor on Treg cells promotes the expansion of these cells, thereby increasing immune regulation (Ohta et al., 2012). In humans 90% of Foxp3^+^ Treg cells also express CD39 however CD73 expression is minimal (Mandapathil et al., 2009; Dwyer et al., 2010; Mandapathil et al., 2010). Antonioli et al. speculated that CD73 may be secreted from human Treg cells and is responsible for the production of adenosine (Antonioli et al., 2013). Qiu et al. demonstrated that cells co-expressing Panx-1 hemichannel and P2Y or P2X7 receptors exposed to high levels of ATP, only transiently activates Panx-1 hemichannel (Qiu and Dahl, 2009). ATP instead of causing a positive feedback loop is actually causing a negative feedback loop and inactivating Panx-1 hemichannel. This mechanism could provide another mode of immuneregulation suppressing the immune response in order to prevent damage caused by prolonged inflammation.

## Purinergic/adenosine receptors and their role in atherosclerosis

Atherosclerosis is a chronic inflammatory disease affecting the vessel wall and a major health issue worldwide (Koupenova et al., 2012a,b). One of the major components of atherosclerosis is the formation of arterial plaques. The progression of atherosclerosis begins with the recruitment of inflammatory monocytes to the area of lipid deposition or arterial injury (Glass and Witztum, 2001; Reiss and Glass, 2006). As discussed in the previous section migration is a Panx-1 hemichannel, purinergic receptor and extracellular ATP dependent process. Infiltrating macrophages in the arterial wall take up large amounts of oxidized low-density lipoprotein (ox-LDL) becoming foam cells loaded with cholesterol (Stary et al., 1994). The accumulation of foam cells leads to the formation of fatty streaks, increase in arterial wall thickness, reduction of oxygen diffusion into the tissue and development of advanced atherosclerosis (Gessi et al., 2010; Hansson and Hermansson, 2011; Moore and Tabas, 2011; Koupenova et al., 2012a,b).

Hypoxia-inducible factor-1 (HIF-1) is a heterodimeric transcription factor comprised of α and β subunits (Wang and Semenza, 1995). HIF-1 adapts cells to low oxygen partial pressure and induces target genes that influence energy metabolism, cell proliferation, hematopoiesis, vascular development, and vasotone (Semenza et al., 1994; Liu et al., 1995; Carmeliet et al., 1998; Kourembanas et al., 1998; Lacombe and Mayeux, 1998; Rose et al., 2002). Zones of hypoxia occur in the atherosclerotic plaque, a result of impaired oxygen diffusion due to the thickness of the lesion, as well as high oxygen consumption by the foam cell. Furthermore, it was demonstrated that ox-LDL induce HIF-1α accumulation in human Mono-Mac-6 (MM6) macrophages (Shatrov et al., 2003). Jiang et al. investigated the gene expression profiles of cultured human U937 cells transfected by HIF-1α-siRNA in response to 24 h of exposure to ox-LDL. Their results indicated that HIF-1α-siRNA inhibits the development of macrophage derived foam cells with ox-LDL by inhibiting the expression of HIF-1α (Jiang et al., 2007). A key function of HIF-1α is the expression of vascular endothelial growth factor (VEGF). VEGF is a regulator of angiogenesis during embryogenesis, skeletal growth and reproductive functions (Ferrara et al., 2003). Together HIF-1α and VEGF are involved in angiogenesis and atherogenesis. As described above the expression of HIF-1α in macrophages under atherogenic conditions promotes the formation of foam cells. Foam cells, macrophages and the U937 myelomonocytic cell line were individually cultured and treated with adenosine under hypoxic conditions which resulted in the accumulation of HIF-1α in all the cells (Gessi et al., 2010). When A1, A2A, A2B, and A3 receptors were knocked down using siRNA there was a reduction in the accumulation of HIF-1α in the cells. In addition, the production of VEGF in foam cells was increased when adenosine was added and strongly reduced with the addition of A2B, and A3 antagonist respectively (Gessi et al., 2010). Hypoxia stabilizes HIF-1α, resulting in the accumulation of adenosine (Gessi et al., 2010). Notably, it can be speculated that in this adenosine-mediated atherosclerosis mechanism, Panx-1 hemichannels may play a role in the release of intracellular ATP into the extracellular environment leading to the formation of adenosine (Figure 1).

Conversely Koupenova et al. determined that the absence of A2B adenosine receptor expression in the liver resulted in a worse atherosclerotic outcome in the double knockout mouse model of apolipoproteinE (ApoE) and A2B adenosine receptor which were fed a high fat/high cholesterol diet (Koupenova et al., 2012a,b). Lack of A2B adenosine receptor led to an elevation of plasma lipids and plaque formation. In this model the liver is responsible for contributing to the anti-atherosclerotic phenotype. Under normal conditions the liver expresses low levels of A2B adenosine receptor. However, with a high fat diet, levels of A2B receptors in the liver increase. Activation of A2B receptor in hepatocytes *in vivo* and *in vitro* causes a decrease in the transcription factor sterol regulatory element binding protein-1 (SREBP-1), which regulates lipid synthesis. Moreover, eliminating A2B adenosine receptor in the liver of the mouse model increased the levels of SREBP-1 and its downstream targets acetyl coenzyme-A carboxylase- α (ACC) and fatty acid synthase (FAS) resulting in upregulation of lipid synthesis. Resulting in the formation of foam cells and the development of atherosclerotic plaques. (Koupenova et al., 2012a,b).

As mentioned above the formation of an atherosclerotic plaque begins with the uptake and accumulation of cholesterol by macrophages and is also influenced by endothelial dysregulation. These atherosclerotic plaques are composed of smooth muscle cells (SMCs), which under normal physiological conditions are found in the medial layer of the artery wall. However, under atherosclerotic conditions SMCs lose their contractile element and gain the ability to replicate and migrate into the intima of the arterial wall (Gorski and Walsh, 1995). Once in the intima SMCs proliferate and begin depositing fibrotic connective tissue (Watson et al., 1998). All of these deregulated cells act as a cover for the fibrous cap that stabilizes the plaque by covering the lipid rich regions. Adenosine and ATP mediate endothelial cell growth, migration, proliferation and death (Burnstock, 2006). ATP binding of P2Y2 and/or P2Y4 stimulates SMC cell proliferation via a mitogen-activated protein kinase (MAPK) cascade contributing to the development of atherosclerosis (Hou et al., 2000). However, adenosine derived from the enzymatic breakdown of ATP by ecto-5′-nucleotidase (see Figure 1) acts as an endogenous modulator protecting against vascular inflammation and immune cell recruitment, therefore, preventing the progression of atherosclerosis (Buchheiser et al., 2011). Adenosine in concert with A2A and A2B receptors has also been shown to stimulate endothelial cell proliferation and regulate the release of platelet-derived growth factor (PDGF) a smooth muscle mitogen from platelets (Jonzon et al., 1985; Adair, 2005).

Moreover, inflammation stimulated by accumulation of ox-LDL in the atherosclerotic plaque activates the release of cytokines and metalloproteinases resulting in degradation of the fibrous cap (Erlinge and Burnstock, 2008). These events result in a weak plaque, which can potentially rupture and release its content into the circulation. This content is highly thrombogenic and produces activation of platelets causing the formation of local thrombus occluding the artery or embolising and resulting in ischemic stroke or myocardial infarction (Erlinge and Burnstock, 2008) Pinheiro et al. demonstrated using human subcutaneous fibroblast that the release of histamine induces an increase in intracellular Ca^2+^ resulting in the release of ATP via Panx-1 hemichannels (Pinheiro et al., 2013). Furthermore, the release of ATP activates P2 receptors and results in fibroblast proliferation and collagen production. The principal cell type of vascular adventitia is fibroblast therefore increase proliferation of this cell type could contribute to atherosclerotic lesion progression and eventual rupture. This evidence suggests a complex mechanism, which results in plaque destabilization, and involves mast cells, histamine, P2 receptors, ATP, Panx-1 hemichannels and fibroblasts.

## Role of Panx-1 hemichannels in apoptosis

There are two main types of cell deaths, apoptosis and necrosis. Morphological features such as cell rounding, DNA fragmentation, externalization of phosphatidyl serine, caspase activation and the lack of an inflammatory reaction characterize apoptosis. Necrosis is characterized by swelling of organelles and plasma membrane, followed by the collapse of the plasma membrane and ending in the uncontrolled release of intracellular contents after the membrane has ruptured which leads to an inflammatory response. Intact apoptotic cells have been shown to release ATP and UTP without extrusion of additional cellular contents, suggesting the opening of relatively large membrane pores such as Cxs or Panx hemichannels during the apoptotic process (Harris, 2007; Elliott et al., 2009; Ghiringhelli et al., 2009; Scemes et al., 2009; MacVicar and Thompson, 2010).

Chekeni et al. showed in Jurkat cells that the channels involved in the release of ATP and UTP in apoptotic cells were Panx hemichannels and not Cxs hemichannels (Chekeni et al., 2010). Inhibition of these channels using 18-alpha-glycyrrhetinic acid (18AGA) or flufenamic acid (FFA), which are efficient Cxs hemichannel blockers had no effect on, the release of ATP induced by intact apoptotic cells (Chekeni et al., 2010). However, when specific Panx hemichannel blockers were used such as probenecid, the release of ATP was blocked from intact apoptotic cells. ATP is a chemoattractant for immune cells thus blocking ATP release by inhibiting Panx-1 hemichannel opening results in a decrease in monocyte recruitment (Chekeni et al., 2010).

Overexpression of Panx-1 increases the release of nucleotides during apoptosis, subsequently increasing monocyte migration. Activation of caspases 3/7 results in opening of Panx-1 hemichannels by a mechanism that involves cleavage of the Panx-1 intracellular carboxy terminal region increasing the release of ATP and UTP which is vital for apoptosis. Using whole cell patch clamp it was determined that opening of Panx-1 hemichannels and subsequent release of ATP occurs in the early events of apoptosis, and no opening of Panx-1 hemichannels was observed in the later events of apoptosis (Chekeni et al., 2010). Sandilos et al. determined that the C-terminus functions as a dissociable channel blocker, capable of inhibiting C-terminally truncated Panx-1 hemichannels and relief of C-terminal inhibition followed by cleavage does not happen if the C terminus is covalently tethered to the channel pore (Sandilos et al., 2012). This evidence suggests a role for Panx-1 hemichannels in the early events of apoptosis.

Divergent from the idea that Panx-1 hemichannels do not form gap junctions Vanden Abeele et al. demonstrated that overexpression of Panx-1 induces formation of Ca^2+^ permeable gap junction channels between cells allowing cellular Ca^2+^ diffusion and facilitating intercellular Ca^2+^ wave propagation (Vanden Abeele et al., 2006). Panx-1 overexpression also increased the Ca^2+^ permeability of the endoplasmic reticulum (ER) membrane and affected intraluminal ER Ca^2+^ concentration. Using human prostate cancer epithelial cells (LNCaP) and human embryonic kidney cells (HEK-293) they demonstrated that while overexpression drastically reduced intraluminal Ca^2+^, endogenous Panx-1 depletion using siRNA increased the content of Ca^2+^ in the ER. This data suggests that Panx-1 hemichannels are not only found on the plasma membrane but also in the ER membrane, and it participates in ER Ca^2+^ leak and intracellular Ca^2+^movement. Vanden Abeele et al speculated that the reduced concentration of Ca^2+^ associated with Panx-1 overexpression could be caused by increase of the BCL-2 family of proteins which plays an important role in the regulation of calcium leak from the ER and is an antiapoptotic protein (Pinton et al., 2000; Vanden Abeele et al., 2002, 2006; Bassik et al., 2004). It may also be due to a deficiency of two pro-apoptotic proteins Bax and Bak (Scorrano et al., 2003; Oakes et al., 2005). This data suggests that Panx-1 could be involved in apoptotic events taking place in endomembranous compartments such as the ER.

## Purinergic receptors and Panx-1 hemichannel importance in HIV-1 infection

The first clinical observations of acquired immune deficiency syndrome (AIDS) were reported in 1981. Since the identification of HIV as the virus responsible for AIDS the countries affected and the numbers of those infected rose to overwhelming numbers. As of 2011, there are 34 million people worldwide living with HIV according to the World Health Organization. A total of 2.5 million new infections were reported in 2011 with 1.7 million deaths related to AIDS.

The established model for HIV entry into cells is mediated by the binding of HIV glycoprotein (gp) 120 to the cellular CD4 receptor. This interaction induces a conformational change to allow the glycoprotein to bind to the co-receptors CXCR4 and/or CCR5. The interaction of gp120 with these two host receptors creates a stable attachment between the virus and the cell membrane facilitating successful viral entry into the cell. In the past two decades studies have documented that binding of HIV or gp120 to the cell rapidly increases the intracellular free calcium concentration (Weissman et al., 1997; Arthos et al., 2000; Liu et al., 2000; Balabanian et al., 2004; Melar et al., 2007). This rapid increase of intracellular free calcium suggests the potential involvement of other membrane receptors or channels in the early stages of HIV infection.

Our laboratory demonstrated that HIV infection of peripheral blood mononuclear cells (PBMCs) and CD^+^_4_ T lymphocytes causes opening of Panx-1 hemichannels in a biphasic manner (Orellana et al., 2013). Binding of the virus to its receptor (CD4) and co-receptors (CXCR4 and/or CCR5) induces opening of Panx-1 hemichannels. Opening of Panx-1 hemichannels in response to the virus resulted in ATP release and subsequent purinergic receptor activation. We also showed that opening of Panx-1 hemichannels was required for HIV entry and replication in CD^+^_4_ T lymphocytes. We propose that opening of Panx-1 hemichannels results in an increase of intracellular calcium and subsequent actin rearrangement, which is a necessary step that allows the virus to fuse with the host cell membrane. The details of these mechanisms are currently under investigation.

**Figure 2:**
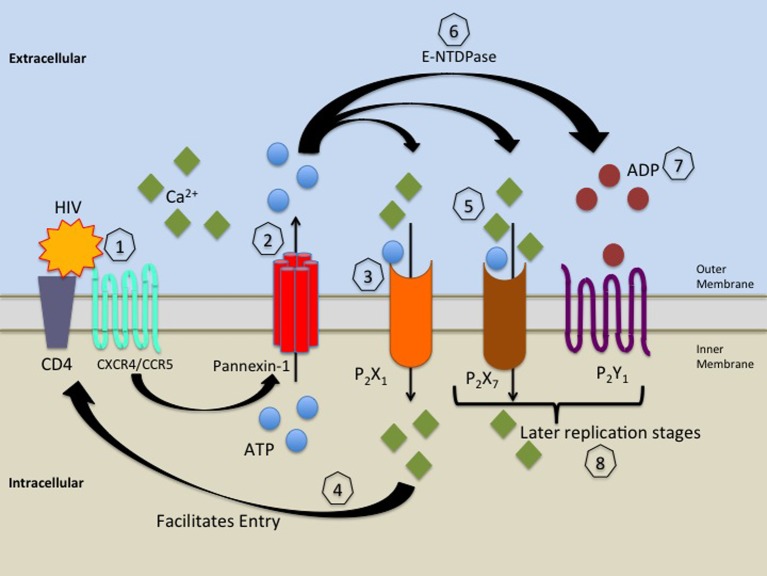
**Proposed model for the role of Panx-1 hemichannels and purinergic receptors in HIV infection**. HIV's binding to CD4 and CXCR4/CCR5 (1) induces signaling which leads to opening of Panx-1 hemichannels and release of ATP (2). Extracellular ATP released through open Panx-1 hemichannels binds to and activates P2X1 receptors causing calcium influx (3), which facilitates HIV entry (4). The release of ATP continues and activates P2X7 and P2Y1 receptors (5), causing further calcium influx inducing downstream signaling, which facilitates later stages of the HIV life cycle (8). Ecto-nucleoside triphosphate diphosphydrolase (E-NTDPase) converts ATP to ADP (6), which activates P2Y1 receptors (7), which increases intracellular calcium, which causes signaling that facilitates later stages of the HIV life cycle (8).

Our laboratory recently described a novel role for purinergic receptors in HIV replication in macrophages (Figure 2). We identified that P2X1, P2X7, and P2Y1 participate in HIV replication. Demonstrating that P2X1 is key in controlling viral entry into human macrophages. Although P2X7 and P2Y1 did not inhibit entry, it is highly likely that these receptors participate in later stages of the viral life cycles (Hazleton et al., 2012). We also identified the gp120's binding to primary human macrophages induces the release of ATP which facilitates autocrine activation of purinergic receptors. Panx-1 hemichannels, purinergic receptors and extracellular ATP play a key role in HIV infection and replication of HIV in immune cells by contributing to entry and possibly in other steps of the viral life cycle. In agreement another study using cell lines and PBMCs indicates that extracellular ATP activates P2Y2 receptors resulting in Pyk2 Kinase activation (Seror et al., 2011). It has been reported that Panx-1 hemichannels, P2Y2 and Pyk2 are physically recruited to the infection synapse (the contact site between the viral and cellular membrane) in order to facilitate infection (Seror et al., 2011). We propose that this process causes membrane depolarization and assists in membrane-to-membrane fusion allowing viral entry.

## Potential hemichannel and purinergic receptor therapies

Purinergic receptor, Panx-1 hemichannel, and extracellular ATP, ADP, AMP, and adenosine blockers and their potential use as therapeutic agents are under current investigation. Commercially available pharmaceutical adenosine, such as adenocard and adenoscan, are currently used to treat supraventricular tachycardia, is an example of an ion channel targeted treatment (Delacrétaz, 2006; Jacobson and Gao, 2006). Other clinically used drugs include dipyridamole and methotrexate, which are used to alter the extracellular adenosine concentration as well as signaling. Currently the US Food and Drug Administration (FDA) approved the A2A receptor agonist regadenoson (Lexiscan; Astellas Pharma) for myocardial perfusion imaging in patients with suspected coronary artery disease (Ghimire et al., 2013).

Among purinergic receptor blockers, which are, consumed daily are food dyes such as Brilliant Blue G (BBG) and Brilliant Blue FCF (BB FCF) which are found in most soft drinks. These dyes are shown to block at least P2X7 receptors, and Panx-1 hemichannels respectively (Jiang et al., 2000; Wang et al., 2013). In addition millions of people worldwide consume caffeine, which antagonize adenosine receptors and is used to treat premature apnoea. Actually, nothing is known of the effect that these dyes and caffeine have in physiological and pathological conditions. For example daily ingestion of these compounds in HIV positive individuals could cause the virus to adapt to these blockers changing the course of the disease. As mentioned above these channels/receptors play a role in inflammation and immune response, therefore individuals who consume these dyes in large quantities could also suffer from a suppressed immune response leading to numerous pathologies and susceptibility to pathogens. Further studies are required to investigate the effect that these dyes have among the human population.

The P2Y12 platelet receptor plays an important role in the genesis of platelet aggregation (Power et al., 2012; Tam et al., 2012). Current treatments blocking adenosine diphosphate (ADP) binding to the P2Y12 receptor, which inhibits platelet aggregation, are commercially available as well as in clinical trials. The first generation thienopyridine drugs which are used for their anti-platelet activity was ticlopidine, which bound irreversibly to P2Y12 platelet receptor (Cattaneo, 2010; Mohelmani and Jackson, 2012). However, its toxicity led to the development of second-generation thienopyridine clopidogrel (Ji and Hou, 2011). Clopidogrel also has its limitations, such as a delay in platelet block because the prodrug requires activation in the liver, and clopidogrel therapy is irreversible which can lead to increase bleeding and transfusion risk in cardiothoracic surgery (Power et al., 2012; Tam et al., 2012). Third generation thienopyridine prasugrel addressed the issue of delayed platelet blocking by being relatively independent of hepatic activation, however, it still remained irreversible and patients were still at risk for increase bleeding (Ferraris et al., 2012). Ticagrelor is an orally administered direct acting platelet blocker, which binds reversibly to the P2Y12receptor. This drug does not require metabolic conversion. It also belongs to a new class of drugs called cyclopyrimidines, which bind, non-competitively to the P2Y12receptor independently of the ADP binding site (van Giezen and Humphries, 2005). Ticagrelor when compared to prasugrel has demonstrated a more promising outcome with fewer side effects.

Numerous clinical trials are on going using various adenosine receptor agonist and antagonist. The expectation are high and could provide treatments for many physiological and pathological conditions such as lipolysis, renal blood flow, immune function, sleep regulation, angiogenesis, inflammatory diseases, ischemia-reperfusion, and neurodegenerative disorders (Sun et al., 2001; Huang et al., 2005; Fredholm, 2007; Johansson et al., 2007; Haskó et al., 2008; Rosenberger et al., 2009; Liu et al., 2010; Eltzschig and Carmeliet, 2011; Eltzschig and Eckle, 2011; Lazarus et al., 2011; Grenz et al., 2012). However, developing adenosine receptor targets is challenging because adenosine signaling is widespread. Therefore it is necessary to use ligands, which could be successfully administered to affect the area of interest, while being safe to use in a clinical setting.

Probenecid is a Panx-1 inhibitor, and has been on the market for decades as a treatment for gouty arthritis. High levels of extracellular potassium ion induce inflammasome activation and caspase 1 cleavage in neurons and astrocytes. In addition probenecid has been shown to attenuate the caspase 1 cleavage in cultured neurons induced by extracellular potassium ions (Peng et al., 2009). Recent evidence using a mouse model has shown that administering probenecid prior to and after stroke induced reduced infarct size, decreased cerebral water content, inhibited neuronal death, and reduced inflammation in the brain (Xiong et al., 2014). These results suggest that probenecid could be used as a treatment for stroke. Another Panx-1 inhibitor is carbenoxolone prescribed to treat oesophageal ulceration and inflammation. Probenecid and carbenoxolone could be ideal candidates as a treatment for pathological as well as physiological conditions were the inhibition of Panx-1 hemichannels could be useful. Other possible treatments could involve the use of mimetic peptides, which are designed with sequences found in the two extracellular loops of the Panx protein. These peptides mimic the loop-to-loop interaction between two hemichannels and activate a docking gate keeping the hemichannel closed. The designs of better and more specific blockers are required in the treatment of diseases involving Panx-1 hemichannels, purinergic receptors and ATP/adenosine.

## Conclusion

In this review we have discussed the role which purinergic receptors and Panx-1 hemichannels play in the pathogenesis of several human diseases. It is crucial to understand the contribution of these receptors and channels in physiological and pathological conditions, in order to design new and improved therapeutic approaches. The contribution that purinergic receptors and Panx-1 hemichannels play in the HIV viral life cycle has only been recently described and unlocking this relationship could hold the key to the development of new preventative therapies and treatments. Purinergic receptor, Panx-1 hemichannel, and extracellular ATP, ADP, AMP, and adenosine are important modulators of many cellular events and hold great potential in understanding and treating many pathological and physiological conditions. The pathologies discussed in this review contribute to a large number of fatalities worldwide. Although much progress has been made in the advancement of treatments for these pathologies, there are still many avenues, which have not been explored. As more information regarding Panx-1 hemichannels and purinergic receptors emerge, the possibility of new therapeutic opportunities for these pathologies emerges as well.

### Conflict of interest statement

The authors declare that the research was conducted in the absence of any commercial or financial relationships that could be construed as a potential conflict of interest.
